# The efficiency of phosphate inactivation in the bottom sediments of small retention reservoirs using a combination of calcium and iron salts, ex situ studies

**DOI:** 10.1038/s41598-025-17779-4

**Published:** 2025-09-24

**Authors:** Lilianna Bartoszek, Piotr Koszelnik

**Affiliations:** Department of Environmental Engineering and Chemistry, Faculty of Civil and Environmental Engineering and Architecture, Rzeszów University of Technology, Al. Powstańców Warszawy 12, 35-959 Rzeszow, Poland

**Keywords:** Internal phosphorus loads, Phosphate inactivation, Calcium and iron salts, Undisturbed sediment cores, Small reservoirs, Ecology, Limnology

## Abstract

In strongly degraded reservoirs, the occurrence of intensive internal supply of phosphate to waters under oxygen-deficient conditions is a problem. The purpose of this study was to evaluate the performance and long-term effectiveness of a properly composed mixture of calcium and iron salts in reducing the intensity of internal supply from the bottom sediments of strongly degraded water reservoirs in terms of their reclamation needs. Experimental studies of phosphate inactivation under anoxic conditions were carried out on undisturbed sediment cores taken from two small retention reservoirs located in SE Poland. The sediments of the tested reservoirs were significantly different in terms of physical and chemical properties. The salt synergy of CaSO_4_∙2H_2_O, FeCl_3_, and CaCO_3_ was ideal to inactivate phosphate phosphorus in the bottom sediments of the tested reservoirs. The application of FeCl_3_ and CaCO_3_ in specific proportions does not lead to a significant increase or decrease in the pH of the water. The decisive influence on the total internal load of phosphorus is the release under dynamic conditions, i.e. during simulated resuspension of sediments and resedimentation of suspended particles. The weaker effectiveness of the applied salt mixture showed in the case of sandy sediments, poor in organic matter, especially humic substances, and significantly hydrated sediments.

## Introduction

One of the most important ecological problems of degraded inland reservoirs is the occurrence of an internal supply of nutrients, the source of which are sediments accumulated on the bottom. The intensity of this supply determines the further intensification of trophic degradation of the reservoirs, especially when measures have been taken to eliminate external loads from the catchment area^1–6^. Protective measures introduced in the catchment area serve their purpose when sediments are not yet a significant source of nitrogen and phosphorus compounds. The occurrence of an intensive internal supply has already conditioned the need for appropriate restoration measures within the reservoir^7,8^. Reclamation of the reservoir aims to stop its degradation and bring it back to a clean-water state, i.e. to force its oligotrophication.

Among the reclamation methods used today, technical, biological, and chemical methods are distinguished^9^. Within technical methods, the most commonly implemented methods are aeration/oxygenation of reservoir waters, removal of hypolimnion waters, and dredging of bottom sediments^8,10,11^. Biological methods mainly include biomanipulation, bioaugmentation, and BIOHYDRO structures, and are currently used as adjunctive treatments for proper reclamation^12,13^. However, the most commonly used methods are chemical methods, i.e. inactivation of phosphorus in bottom sediments and capping, i.e. coating the sediment surface with a reactive material that blocks the release of phosphate^7,9,10,14,15^. For the inactivation of phosphorus in sediments, iron, aluminium, and calcium salts are most often used, i.e., metals that in the environment form hardly soluble chemical compounds with phosphorus that precipitate as sediment^13,16,17^. Iron compounds in the form of Fe_2_(SO_4_)_3_, FeCl_3_, or the coagulant PIX-112 (based on Fe_2_(SO_4_)_3_), due to their lower effectiveness in oxygen-deficient conditions, are currently most often used in parallel with aeration or oxygenation of the bottom zone^12,18–21^. Aluminium compounds are applied in the form of Al_2_(SO_4_)_3_ or the coagulant PAX18 (an aqueous solution of polyaluminium chloride) and have shown good efficiency in blocking the internal supply of phosphate regardless of oxygen conditions. However, as a result of the ageing of the coagulant and due to the alkalinisation phenomenon of the waters in eutrophic reservoirs, there is a danger of the release of toxic ionic forms of aluminium into the water (pH > 8)^11,14,22–24^. To limit this, aluminium salts are applied in small doses at a high frequency or continuously for up to a dozen years^25–27^. Lanthanum-modified bentonite clay, commercially known as Phoslock, has shown the highest effectiveness in phosphate inactivation regardless of the levels of water oxygenation and pH. The disadvantages are the high economic costs of the reclamation and the observed bioaccumulation of the toxic form of lanthanum (La^3+^) in fish and crayfish organisms after the reclamation of the lake^15,18,22,28^. Various metal/material combinations are also currently being tested, e.g., the "flock and lock" technique, in which natural material is used with the addition of P-bonding metal salts (most commonly La or Al) to improve phosphorus binding in the sediment and at the same time cause sedimentation/flocculation of algae from the water column^8,11,13,29^. Of the calcium compounds, Ca(OH)_2_ has been used individually or to reduce reclamation costs in combination with CaCO_3_, mainly in lakes in Canada, but also in Australia, the USA and Europe. Calcium hydroxide is more effective, but an increase in water alkalinisation may be associated with its application. No increase in pH above 9.5 has been observed with repeated applications of small amounts of this substance^20,21,27^. Calcium sulphate dihydrate (VI) has been used in the reclamation of a Finnish lake^30^. Studies conducted on the sediments of the Solina and Myczkowce dam reservoirs have shown that CaSO_4_∙2H_2_O effectively increases the retention of phosphorus in the bottom sediments characterised by an adequate iron content^31,32^. This is because the use of gypsum in phosphorus inactivation under oxygen deficiency conditions involves the risk of releasing H_2_S, which is toxic to aquatic organisms^33^. However, sulphates are commonly used in the chemical reclamation of lakes in the form of Fe_2_(SO_4_)_3_ or Al_2_(SO_4_)_3_^17,20^. Abiotic factors such as pH, oxygenation, and redox potential must be taken into account when choosing the most suitable material for the chemical reclamation of water reservoirs^7,34^.

The use of phosphate binding agents in lake reclamation has been practised by lake managers for several decades. On a technical scale, aluminium or lanthanum compounds/preparations are the most widely used^27,35–37^. Lanthanum preparation has been used successfully in many lakes in Europe, the United States, Canada, New Zealand, and Australia, across a wide range of morphologies and destinations^20,21,27,35,38^. Positive reclamation effects following the application of aluminium compounds have been confirmed in lakes (Długie, Starodworskie, Barleber) even after 15–20 years ^36,39,40^. Currently, two approaches are used in Polish reservoirs. The first is the so-called sustainable reclamation involving pro-ecological methods, i.e. the application of small doses of iron (III) sulphate several times a year together with aeration and supportive biomanipulation (e.g., Lake Durowskie, Malta Reservoir)^41,42^. The second approach is the sequential application of polyaluminium chloride and iron (III) chloride (Fe-shallow water zone, Al-deep water zone) (e.g., Lake Mielenko, Klasztorne Małe) ^43,44^. Many different geoengineered materials (synthetic and modified minerals) with promising sorption properties are currently being developed and tested in laboratories^9,15,18,22,45,46^. Most of them may never be implemented on a technical scale, especially in large-scale applications, due to the high economic cost, complex production process, lack of research on potential side effects, and in situ trials.

The purpose of this study was to evaluate the performance and long-term effectiveness of a properly composed mixture of calcium and iron salts in reducing the intensity of internal supply from the bottom sediments of strongly degraded small water reservoirs in terms of their reclamation needs. In strongly degraded reservoirs, the problem is the occurrence of an intensive internal supply of phosphate to the waters under oxygen deficit conditions^47^. According to estimates, there are more than 16 million small water bodies in the world^48^. Due to their low resilience (low capacity and depth) and the usually strong anthropogenic pressure of the catchment areas, they are largely degraded objects^49^. This disqualifies them as drinking water resources as well as recreational uses. Therefore, more research is needed in the search for and testing of low-cost reactive materials based on various substances found in the natural aquatic environment to effectively eliminate the internal supply of phosphates and further advance trophic degradation in small water reservoirs. The proposed mixture of calcium and iron salts was refined in terms of composition and proportions on the basis of laboratory experiments (Research Project). The tests carried out under these conditions indicate that it has a high potential to inhibit the internal supply of phosphates from sediments and thus stop the progression of trophic degradation of water reservoirs. Effective blocking of internal phosphate loads in the bottom sediments and, at the same time, not adversely affecting the pH of the water, thereby damaging the biocenosis of the reclaimed reservoir, is important for the proper functioning of the aquatic ecosystem.

## Materials and methods

### Study area and sampling strategy

**Fig. 1:**
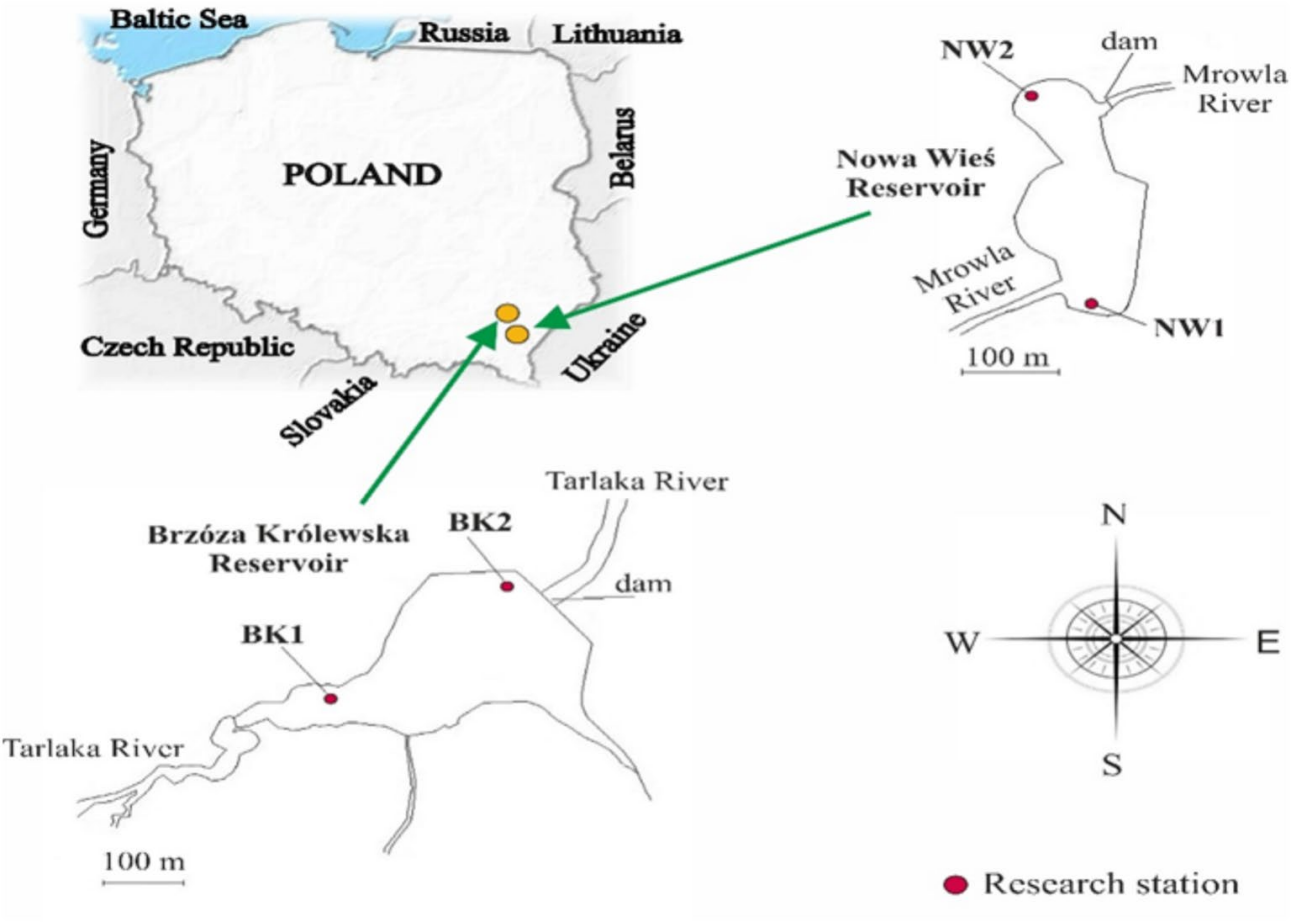
Location of sampling stations on the Brzóza Królewska and Nowa Wieś reservoirs.

The studies were carried out on undisturbed sediment cores taken from two sites (near an inflow and in the dam area) at two small retention reservoirs located in SE Poland (Fig. 1). The most important parameters of the reservoirs are shown in Table 1. The Nowa Wieś reservoir is a small retention reservoir located in suburban areas. The river basin that feeds the reservoir is used intensively for agriculture and in densely urbanised areas. The reservoir has never been reclaimed. The Brzóza Królewska reservoir is located in a rural area with a typical forest-agricultural catchment with buildings. It is mainly exposed to anthropopressure related to agriculture and livestock agriculture.

**Table 1:** Selected parameters of reservoirs and their catchments.

Parameters	Brzóza Królewska	Nowa Wieś
Year of construction	1978	1977
Coordinates	50°14’N 22°19’E	50°06’N 22°03’E
Total capacity [10^3^ m^3^]	50	75
Total area [ha]	7.05	3.0
Max depth (mean) [m]	1.5 (0.7)	3.0 (1.0)
Mean retention time [d]	2.5	1.3
Catchment area [km^2^]	30.4	208.1

### Experimental studies

Laboratory tests of the effectiveness of the tested salt mixture under anoxic conditions (i.e. total lack of dissolved oxygen) were carried out at 2-h, 24-h and 10-week exposures. Undisturbed sediment cores were collected in the summers of 2014 and 2015 using a pipe dipper (KC Kayak, Denmark) directly into 6 Plexiglass tubes (0–5 cm layer) of 33.5 cm length and 5.1 cm inner diameter. The tubes were closed from the bottom with rubber plugs. The supernatant solutions were prepared by diluting the reservoir water 1:10 with distilled water. Anoxic conditions were obtained by deoxygenating the supernatant solutions with anhydrous sodium sulphate (IV). The solutions prepared in this way contained approximately zero concentration of phosphate phosphorus, that is, in the range of 0.004 to 0.057 mgP∙dm^-3^. The salt mixture tested consisted of calcium sulphate (VI) dihydrate (CaSO_4_∙2H_2_O), iron (III) chloride (FeCl_3_) and calcium carbonate (CaCO_3_). The mass ratio of Ca:Fe in the mixture was approximately 2.5:1. For the Brzóza Królewska reservoir, the mixture doses used were 0.09–0.23 g (2 h exposure), 0.14–0.28 g (24 h) and 0.18–0.32 g (10 weeks). Predicting a much higher phosphorus load released from the sediment for the Nowa Wieś reservoir, higher doses of the inactivation mixture were designed in the range of 0.28–0.41 g (2 h), 0.37–0.51 g (24 h), and 0.41–0.69 g (10 weeks). The salt application was carried out directly on the sediment surface. The previously prepared solution was then introduced with a small stream along the walls of the tubes (leveraging). The sediment cores were resuspended by stirring a surface layer of about 1 cm using a mechanical stirring device (about 150 rpm). After ten minutes of stirring, the samples were left under constant thermal conditions (14–16 °C) without light for the remaining exposure time. After the scheduled exposure time, the concentration of phosphate phosphorus in the supernatant solutions was determined by the spectrophotometric method with ammonium molybdate and ascorbic acid (Aquamate spectrophotometer, Thermo Spectronic). After removing the supernatant solution, the sediment cores (after a 10-week exposure) were flooded again with a deoxygenated solution with zero phosphate phosphorus concentration (without reapplication of the salt mixture) and subjected to resuspension (so-called random) to verify the stability of phosphate inactivation^49^.

### Analysis of sediment parameters

Bottom sediment samples (0–5 cm layer) for parameter analysis were collected at the same locations during the spring–autumn period in 2013–2014 (nine samples at each site). The water content was determined by weight by drying to constant weight at 105 °C. The proportion of sediment granulometric fractions was determined using the sieve-areometric method^50^. The sediments for analysis were dried (60 °C for 48 h) and homogenised. The organic matter (OM) content of the sediment was calculated based on the loss on ignition (550 °C for 4 h). The content of humic substances (HS) was determined using the modified Griffith-Schnitzer method^50^. Phosphorus fractionation was performed in sediments using the Standards, Measurements and Testing (SMT) method. This procedure allows three fractions to be separated by extraction: non-apatite inorganic phosphorus (NAIP), i.e. P in compounds with Fe, Mn and Al, apatite phosphorus (AP), i.e. P in compounds with Ca and organic phosphorus (OP)^51^. The sediments were subjected to mineralisation in concentrated HNO_3_ and at high pressure (2–4.5 MPa) in a microwave mineraliser (UniClever II, Plazmatronika, Poland). In the digestion solution, total phosphorus (P_tot._) was determined spectrophotometrically as phosphates, as well as Fe, Mn, Al and Ca contents. These metals were determined using an ICP spectrometer (Integra, GBC). The parameters used to characterise the sediments of the studied reservoirs were discussed in more detail in another paper^51^.

### Statistical analysis

An analysis of variation in the size of the variables was performed for the selected parameters. For this purpose, the Mann–Whitney U test (comparison of means in two groups), Fisher-Snedecor test and Kruskal–Wallis test (evaluation of differences between means in several groups) were used. The probability of error in accepting the hypothesis of the existence of differences between the tested means was taken at the 5% level (p = 0.05). Calculations were performed with Statistica 13.0 PL (StatSoft Poland).

## Results and discussion

### Efficiency of phosphorus inactivation in sediments

**Fig. 2:**
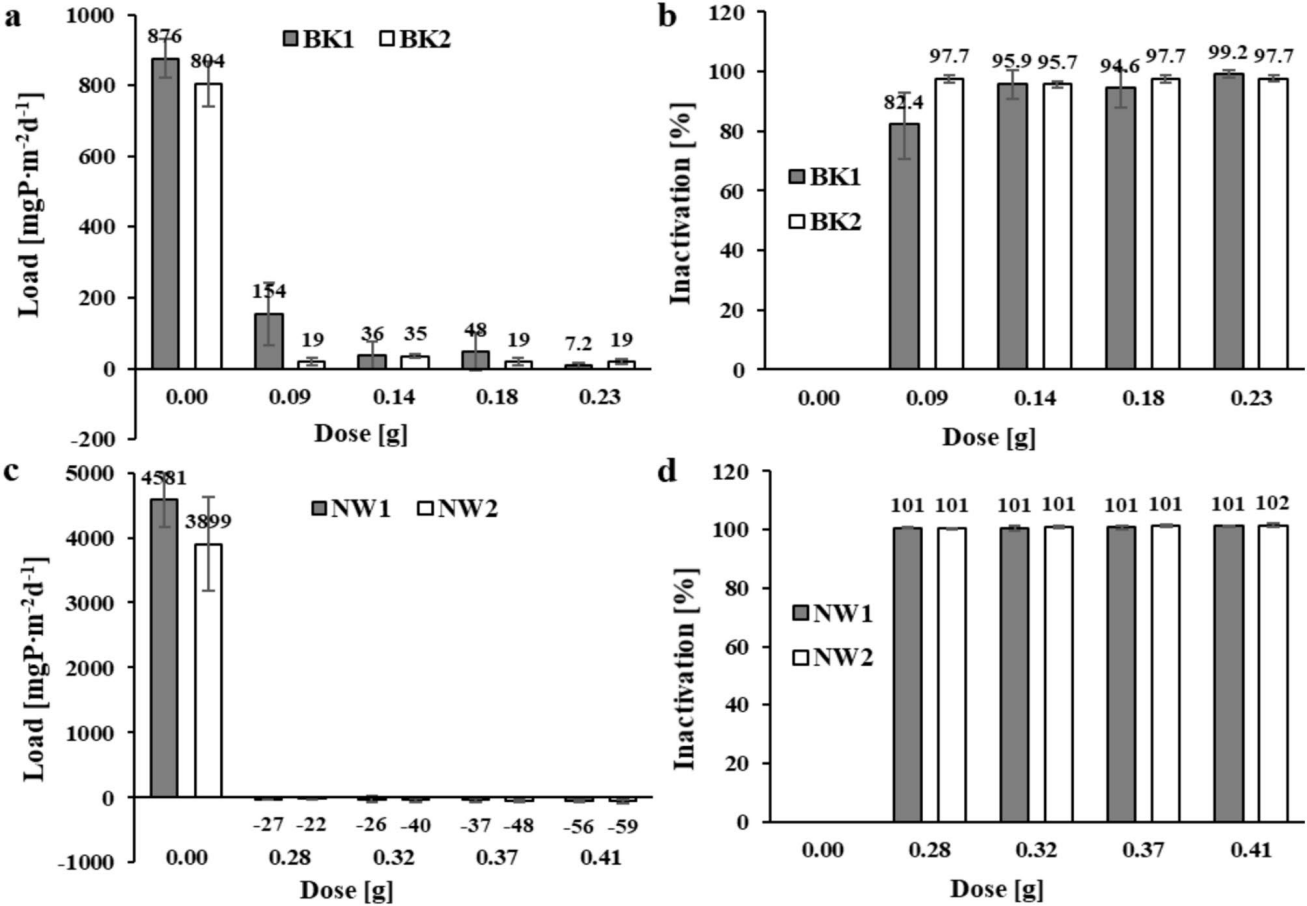
The amount of phosphate phosphorus load released and inactivation in reservoir sediments after 2 h of exposure: (**a**) and (**b**) – Brzóza Królewska, (**c**) and (**d**) – Nowa Wieś (respectively).

For Brzóza Królewska reservoir sediments, the initial concentration (C_p_) of phosphate phosphorus in solution occurred at 0.004–0.005 mgP∙dm^-3^. In blank samples (without mixture dose) after 2 h of exposure, phosphate phosphorus concentration increased (with respect to C_p_) by an average of 0.304 mgP∙dm^-3^ for sediments near the tributary (BK1) and by 0.279 mgP∙dm^-3^ for sediments in the dam area (BK2). When converted to sediment area and day, an internal load of 876 and 804 mgP∙m^-2^d^-1^ was obtained (for BK1 and BK2, respectively) (Fig. 2a). In samples to which the salt mixture was applied, significantly lower internal loads in the range of 7–154 mgP∙m^-2^d^-1^ were obtained. High inactivation efficiencies of 95.9% and 97.7% were already obtained with a dose of 0.14 g and 0.09 g (respectively) for BK1 and BK2 (Fig. 2b). There was no statistically significant effect of doses of 0.14–0.23 g of the mixture on the amount of phosphate release from the sediments of this reservoir under anoxic conditions.

The significant enrichment in total phosphorus and its fractions and organic matter of the sediments of the Nowa Wieś reservoir suggested that the sediments of this reservoir under anoxic conditions could be an important source of internal supply ^51^. Due to the more polluted water in this reservoir, the initial concentration (C_p_) of phosphate phosphorus was 0.028–0.035 mgP∙dm^-3^. In the blank samples, after 2 h of exposure under anoxic conditions, an increase in the concentration of phosphate phosphorus in solution was obtained by an average of 1.59 mgP∙dm^-3^ for sediments near the tributary (NW1) and 1.35 mgP∙dm^-3^ for sediments collected in the dam area (NW2). The amount of internal load in the blank samples was therefore 4581 and 3899 mgP∙m^-2^d^-1^ (for NW1 and NW2, respectively) (Fig. 2c). In anticipation of a much higher phosphorus load released from these sediments, higher doses of the inactivating mixture were planned, which proved to be sufficient to achieve a complete block of phosphate phosphorus released from the sediments. In the samples to which the salt mixture was applied, internal loads were obtained in the range of negative values (from –22 to –59 mgP∙m^-2^d^-1^), which means that in addition to inactivation of the released loads, there was also retention of phosphorus present in solution before the experiment (C_p_). Phosphorus inactivation of 100% (100.6%) was already achieved at a mixture dose of 0.28 g (Fig. 2d). Increasing the dose (> 0.32 g) had no statistically significant effect on the amount of phosphate release from the sediments of this reservoir under anoxic conditions.

**Fig. 3:**
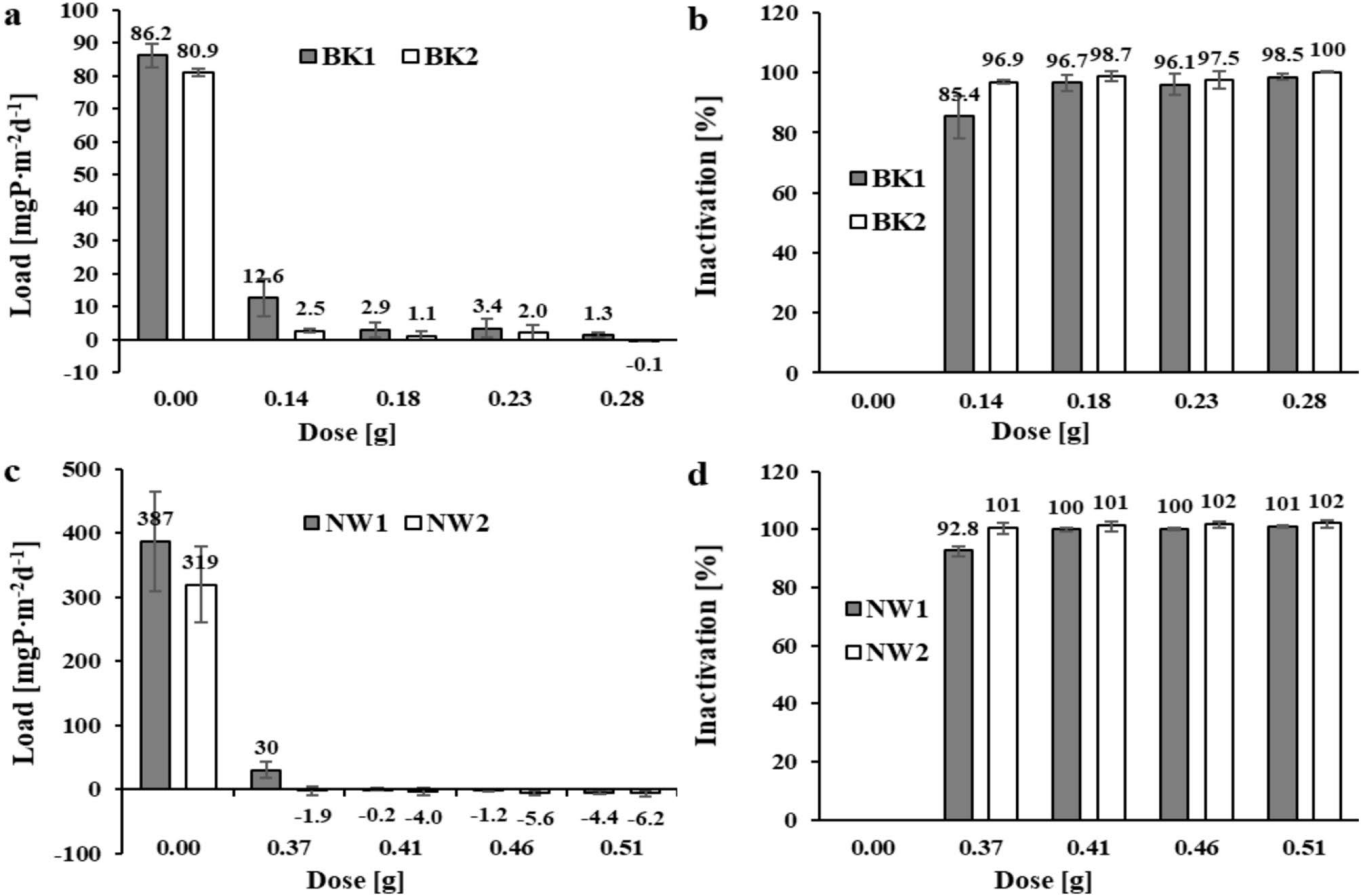
The amount of phosphate phosphorus load released and inactivation in reservoir sediments after 24 h of exposure: (**a**) and (**b**) – Brzóza Królewska, (**c**) and (**d**) – Nowa Wieś (respectively).

In anticipation of a higher release during 24-h exposure under anoxic conditions, slightly higher doses of the salt mixture were used. The concentration of phosphate phosphorus in the supernatant solutions of the blank samples from the Brzóza Królewska reservoir increased by 0.359 and 0.337 mgP∙dm^-3^, which corresponds to 86.2 and 80.9 mgP∙m^-2^d^-1^ (Fig. 3a). In samples with the salt mixture, the internal loads ranged from –0.1 to 12.6 mgP∙m^-2^d^-1^. Using 0.18 g of the mixture, inactivation levels of 96.7 and 98.7% were achieved (Fig. 3b). There was no statistically significant effect of higher doses (0.23 and 0.28 g) of the salt mixture on the amount of phosphate release from the sediments of this reservoir under the experimental conditions given.

After 24 h of exposure of the Nowa Wieś reservoir sediments under anoxic conditions in the solution of the blank samples, the concentration of phosphate phosphorus increased by an average of 1.61 and 1.33 mgP∙dm^-3^. The internal load of phosphorus released in the blank samples was 387 and 319 mgP∙m^-2^d^-1^, respectively (Fig. 3c). In samples with the salt mixture, the internal phosphorus loads partially assumed negative values of –6 to 30 mgP∙m^-2^d^-1^, indicating that the phosphate phosphorus contained in the solution before exposure (C_p_ 0.023–0.057 mgP∙dm^-3^) was also immobilised in the sediment. The application of 0.37 g of the salt mixture resulted in a phosphorus immobilisation of 92.8 and 100.7% (Fig. 3d). Another 0.41 g applied dose applied immobilisation of phosphorus at 100.1% and 101.4%. There was no statistically significant effect of higher doses of the mixture (0.46–0.51 g) on the amount of phosphate release from the sediment of this reservoir after exposure for 24 h under anoxic conditions.

**Fig. 4:**
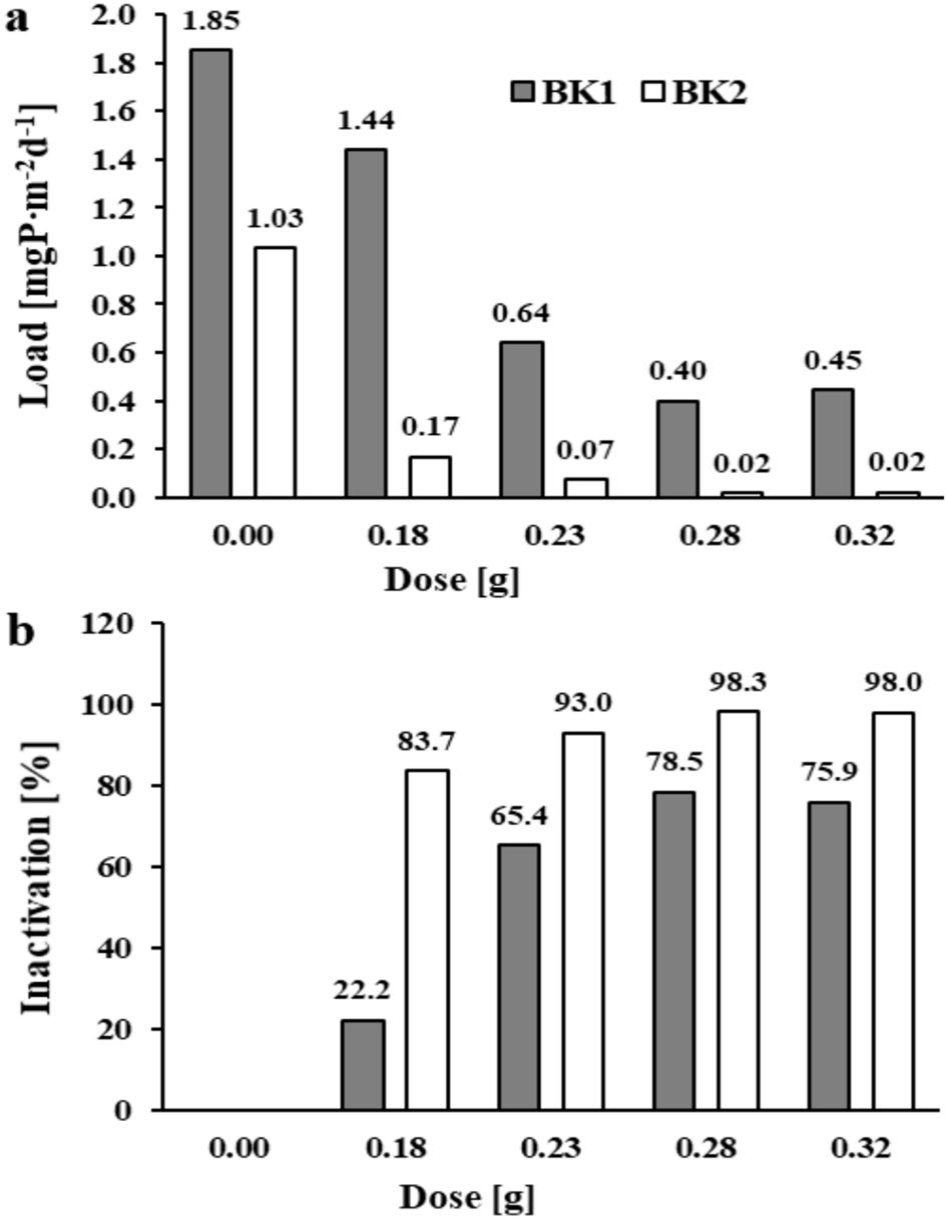
The amount of phosphate phosphorus load released (**a**) and inactivation (**b**) in the sediments of the Brzóza Królewska reservoir after 10 weeks of exposure.

Exposure at 10 weeks under anoxic conditions was intended to demonstrate the effectiveness of phosphorus inactivation with the salt mixture in the long term. In blank samples after 10 weeks of exposure of the Brzóza Królewska reservoir sediments, an increase of 0.540 and 0.301 mgP∙dm^-3^ (BK1 and BK2, respectively) in phosphate phosphorus concentration was obtained in the supernatant solution. The internal loads of phosphorus released into the solution in the blank samples were 1.85 and 1.03 mgP∙m^-2^d^-1^ (BK1 and BK2) (Fig. 4a). In samples with the salt mixture, the internal loads ranged from 0.40–1.44 and 0.02–0.17 mgP∙m^-2^d^-1^ for the sediments of the respective sites. At a dose of 0.18 g of the mixture, inactivation of 22.2 and 83.7% (BK1 and BK2, respectively) was obtained (Fig. 4b). Doses of 0.28 and 0.32 g of the mixture allowed inactivation of phosphorus in the range of 75.9–78.5% in sediments collected near the tributary (BK1) and 98.0–98.3% in sediments from the dam area (BK2).

**Fig. 5:**
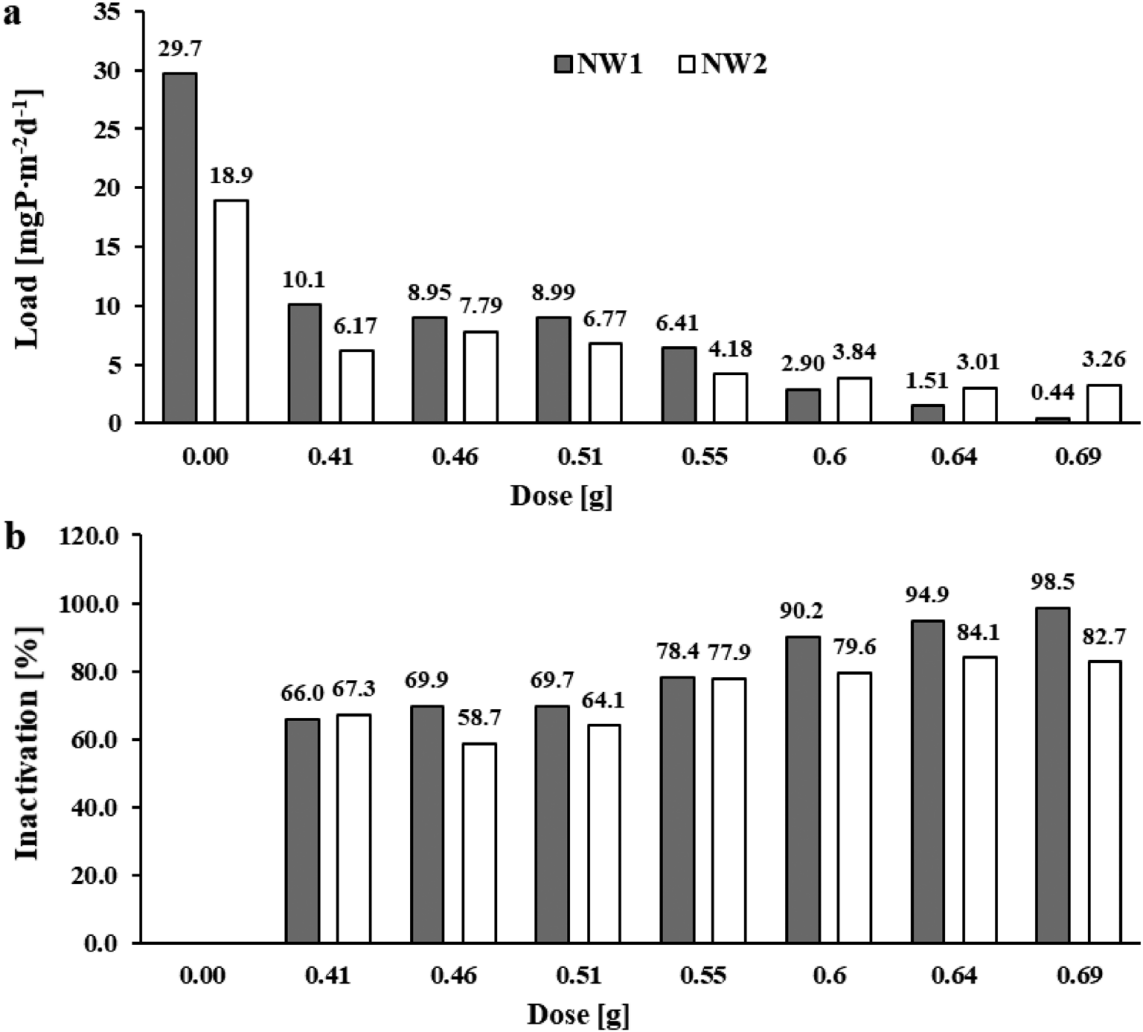
The amount of phosphate phosphorus load released (**a**) and inactivation (**b**) in the sediments of the Nowa Wieś  reservoir after 10 weeks of exposure.

In the case of the Nowa Wieś reservoir sediments, phosphate phosphorus concentrations in the solutions of the blank samples increased by 8.65 and 5.50 mgP∙dm^-3^ (for NW1 and NW2, respectively) after 10 weeks of exposure. This corresponded to an internal phosphorus load of 29.7 and 18.9 mgP∙m^-2^d^-1^ (Fig. 5a). However, in the samples with the mixture, the phosphorus load released from the sediments was in the range of 0.44–10.1 mgP∙m^-2^d^-1^ (NW1) and 3.01–7.79 mgP∙m^-2^d^-1^ (NW2). Only with the application of 0.64 and 0.69 g of the salt mixture was phosphorus inactivation achieved at 94.9 and 98.5% for sediments near the tributary (NW1) and 84.1 and 82.7% for sediments in the dam area (NW2) (Fig. 5b).

## Stability of phosphorus inactivation in sediments

**Fig. 6:**
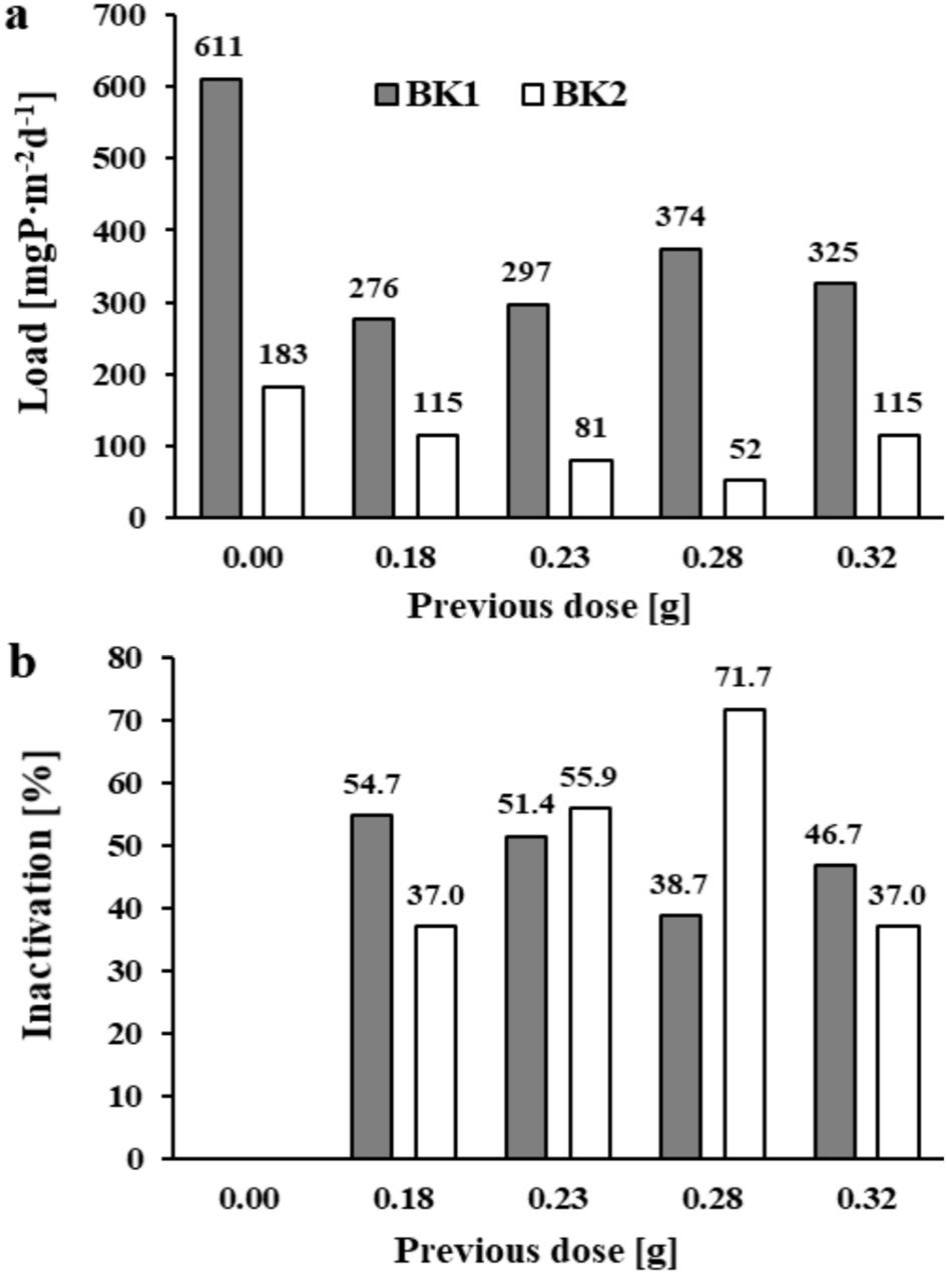
The amount of phosphate phosphorus load released (**a**) and inactivation (**b**) in the sediments of the Brzóza Królewska reservoir after repeated resuspension and 2 h of exposure (without reapplication of the salt mixture).

**Fig. 7:**
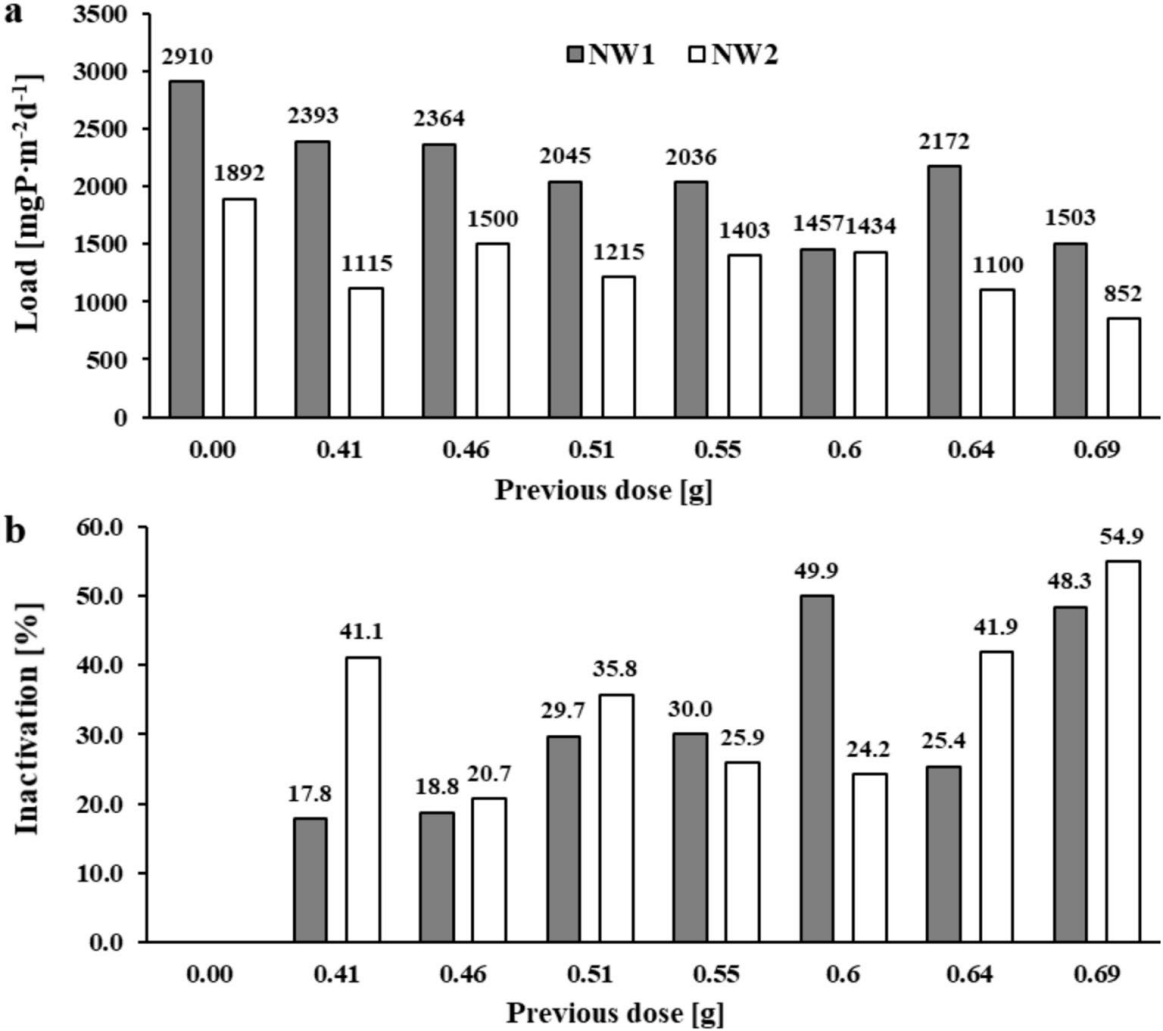
The amount of phosphate phosphorus load released (**a**) and inactivation (**b**) in the sediments of the Nowa Wieś reservoir after repeated resuspension and 2 h of exposure (without reapplication of the salt mixture).

The sediment cores of both reservoirs were reexposed for 2 h (including 10 min of resuspension) under anoxic conditions after changing the supernatant solution and without reapplication of the salt mixture. The test was to show whether accidental disturbance of the sediments, e.g. wind resuspension, would initiate further phosphate release. For Brzóza Królewska reservoir sediments, an increase of 0.212 and 0.064 mgP∙dm^-3^ (BK1 and BK2) was obtained in the solutions of the blank samples. This corresponded to an internal phosphorus load of 611 and 183 mgP∙m^-2^d^-1^ (Fig. 6a). In contrast, the sediments to which the salt mixture had been applied 10 weeks earlier still released a fairly substantial phosphorus load in the range of 276–374 and 52–115 mgP∙m^-2^d^-1^. This corresponded to an inactivation in the range of 38.7–54.7% for BK1 and 37.0–71.7% for BK2 (not always according to the dose of the mixture) (Fig. 6b). In blank sediment samples from the Nowa Wieś reservoir, after repeated 2-h exposure, the concentration of phosphate phosphorus in solution increased by 1.01 and 0.657 mgP∙dm^-3^ (NW1 and NW2). This corresponded to internal loads of 2910 and 1892 mgP∙m^-2^d^-1^ (Fig. 7a). In the remaining samples, despite the earlier application of the mixture, significant phosphorus loads were recorded in the range of 1457–2393 and 852–1500 mgP∙m^-2^d^-1^ (with frequent deviations). A higher inactivation of phosphorus of approximately 50% was achieved for samples to which the highest dose of the mixture, 0.69 g, had been previously applied (Fig. 7b).

## Phosphorus release depending on sediment composition and exposure conditions

The sediments of the studied reservoirs differed in their granulometric and chemical composition (Table 2). The sediments of the Brzóza Królewska reservoir were clearly distinguished from those of the Nowa Wieś reservoir by the dominant share of the sand fraction (85% on average) and the minimal content of the clay fraction (1.24%). The sediments of the Nowa Wieś reservoir are sandy–silty sediments (40% sand and 50% silt) with a small amount of clay (approximately 10%). Statistical analysis showed that the sediments of the studied reservoirs differed significantly between themselves (reservoir average) in terms of the content of OM, P_tot._, NAIP, AP, OP, Fe, Mn, Al, Ca (p < 0.001). At the same time, the sediments of the Nowa Wieś reservoir were highly enriched in total phosphorus and its fractions, organic matter and metals that form insoluble connections with phosphorus. However, the sediments of the Brzóza Królewska reservoir were characterised by low contents of the mentioned parameters, typical of sandy sediments ^51^. The sediments of the Brzóza Królewska reservoir near the tributary were slightly more contaminated, which is also typical of most small water reservoirs ^49,50^.

**Table 2:** Selected parameters in the bottom sediments of reservoirs in 2013–2014, based on ^51^ (Average ± SD, except grain size). OM–organic matter, P_tot._–total phosphorus, OP–organic phosphorus, NAIP–non-apatite, inorganic phosphorus (Fe–P, Al-P, Mn-P), AP–apatite phosphorus (Ca-P), HS–humic substances.

Parameters	BK1	BK2	NW1	NW2
Sand [%]	77.8	92.9	40.8	38.8
Silt [%]	19.8	7.06	49.9	50.2
Clay [%]	2.46	0.01	9.26	11.0
Water content [%]	58.1 ± 8,9	26.8 ± 4,7	65.3 ± 6,1	70.7 ± 4,5
OM [%]	6.48 ± 3.73	1.00 ± 0.51	12.4 ± 4.2	14.0 ± 2.7
P_tot._ [mg∙g^-1^ dw]	0.514 ± 0.429	0.077 ± 0.023	1.96 ± 0.91	2.60 ± 0.64
OP [%]	20.8 ± 9.4	29.6 ± 17.3	13.0 ± 2.3	12.5 ± 2.0
NAIP [%]	60.7 ± 12.3	52.0 ± 16.5	68.8 ± 4.3	71.1 ± 5.4
AP [%]	9.24 ± 3.28	17.4 ± 6.3	18.8 ± 2.2	16.9 ± 3.7
Fe [mg∙g^-1^ dw]	7.44 ± 4.39	2.48 ± 0.95	26.7 ± 9.5	33.7 ± 3.2
Mn [mg∙g^-1^ dw]	0.261 ± 0.164	0.091 ± 0.024	0.628 ± 0.215	1.68 ± 0.73
Al [mg∙g^-1^ dw]	7.19 ± 3.85	2.47 ± 0.53	23.5 ± 8.5	33.6 ± 4.2
Ca [mg∙g^-1^ dw]	2.41 ± 1.40	0.813 ± 0.166	13.1 ± 3.2	14.2 ± 3.6
HS [mgC∙g^-1^ dw]	28.8 ± 17.0	2.78 ± 1.76	37.9 ± 14.2	39.4 ± 8.3

**Fig. 8:**
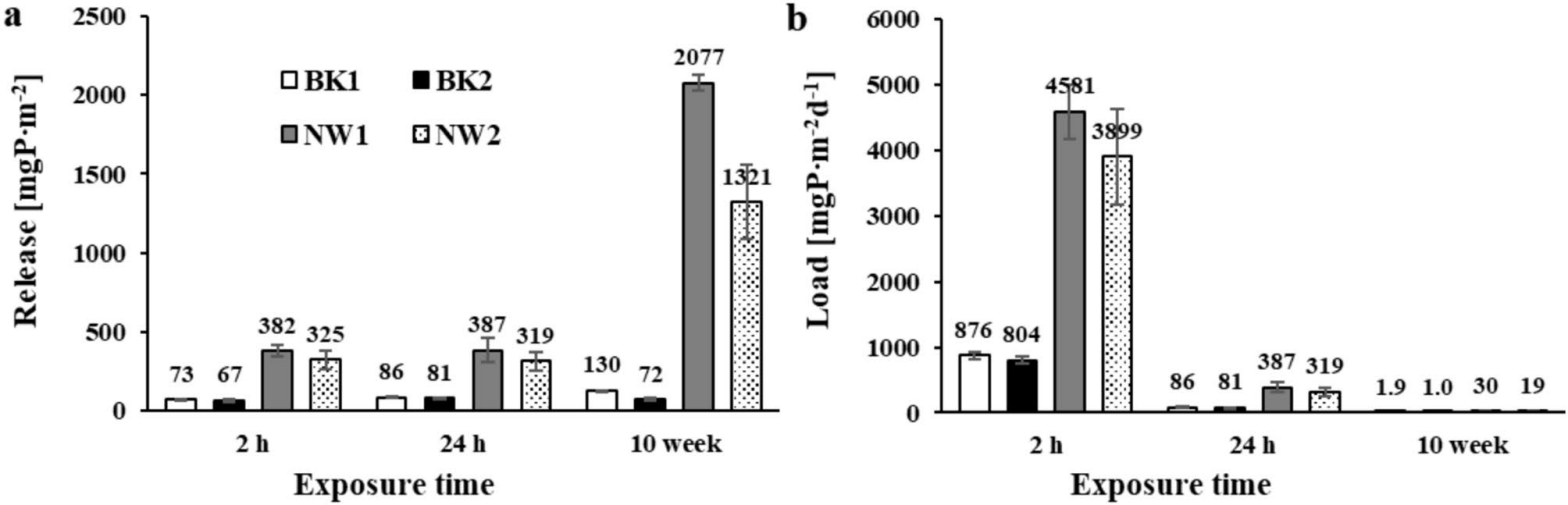
The amount of phosphorus flux [mgP∙m^-2^] (**a**) and internal loads [mgP∙m^-2^d^-1^] (**b**) released without the application of the salt mixture (blank samples) for the sediments tested and different exposure times.

In samples without application of salt mixture for the sediments of the Nowa Wieś reservoir (Fig. 8a), several to even a dozen times higher amounts of phosphorus released (in mgP∙m^-2^) were observed compared to the sandy sediments of the Brzóza Królewska reservoir. Increasing the exposure time to 24 h did not have a significant effect on the amount of phosphorus release. Although slightly higher phosphorus release was found for BK1, BK2, and NW1, the situation was reversed for NW2. Statistical analysis did not show significant variation in the amount of phosphate release (in mgP∙m^-2^) from the sediments of the studied reservoirs between 2 and 24 h exposure times. However, after 10 weeks of exposure, the amount of phosphorus released was significantly higher in the case of the Nowa Wieś reservoir (several times) and Brzóza Królewska near the tributary (BK1). The release of phosphate (in mgP∙m^-2^) from the sediments was statistically significantly (p < 0.05) higher than that obtained after 2 and 24 h of exposure. When the internal load (mgP∙m^-2^d^-1^) was calculated, the trend appeared to be downward with increasing exposure time (Fig. 8b). This indicates that release under dynamic conditions, i.e. during simulated sediment resuspension and suspension sedimentation, has a decisive effect on the total internal phosphorus load. Calculated phosphorus loads (mgP∙m^-2^d^-1^) after 2 h of exposure represent a possible supply under extreme environmental conditions, ie, frequent resuspension of the surface sediment layer (in ex situ studies 10 min/2 h) and continuous depletion of phosphate as a result of assimilation by intensively growing vegetation. During 24-h exposure, sediment resuspension occurred only once (10 min/24 h) and after sedimentation of the suspension, further phosphorus release had already occurred under static conditions, hence the small differences between the amounts of phosphorus released (Fig. 8a). After 10 weeks of exposure, a higher amount of released phosphorus was obtained (in mgP∙m^-2^), but when converted to daily loads, they turned out to be very low (Fig. 8b). Here, too, resuspension and sedimentation were carried out only once. During the rest of the exposure time, the release of phosphate occurred under static conditions. The oxidation and reduction processes that take place and the depletion of electron acceptors intensified the flux of released phosphorus also from inorganic connections. The frequency and intensity of sediment resuspension in shallow water reservoirs depends on hydrological conditions, that is, the velocity of water flow and the degree of shoreline sheltering from the direction of prevailing winds^52^. According to Abesser and Robinson^53^, wind speeds above 13 m∙s^-1^ can already generate resuspension and deposition of sediment particles at depths of up to 20 m and potentially disturb sediment in large parts of the reservoir.

## Analysis of the activity of the salt mixture

Previous studies have shown that the application of gypsum to water increases the sedimentation rate of suspended particles without reducing the amount of phosphorus retained by sedimenting sediments^32^. In anoxic sediments, SO_4_^2-^ can be used as an electron acceptor in the oxidation of organic matter by sulphate-reducing bacteria. The H_2_S produced binds to Fe^2+^ to form FeS ^28,33,54^. If iron is not present in the sediment in sufficient amounts, the sulphides will not precipitate, and hydrogen sulphide, which is harmful to aquatic organisms, will be released into the water column. The formation of iron (II) sulphide and pyrite can, in turn, interfere with the formation of iron (II) phosphate (V) under anoxic conditions^28,33,55^. In addition, reactions of S (II) with metals can lead to the release of oxyanions bound to metal oxides^56^. Therefore, gypsum application will usually involve the introduction of additional iron compounds. The application of ferric (III) chloride is associated with a decrease in the pH of the water^28,45^, so it becomes necessary to correct the pH with CaCO_3_. Calcium carbonate (in stoichiometric amounts) neutralises hydrochloric acid formed and reduces the negative effects of FeCl_3_ on the aquatic environment. In contrast, too high a pH will hinder phosphate binding on sorbents used in P immobilisation due to competition with hydroxyl ions for binding sites^34^. Measurements during the study showed that the pH of the supernatant solutions was in the range of 7.14–7.90 in the blank samples and 6.76–7.27 in the samples with the addition of the salt mixture. The mechanism of activity of the salt mixture may involve the adsorption of phosphate onto calcium carbonate and iron (III) hydroxide particles (less durable bonding) and/or the formation of calcium and iron phosphates. Calcium phosphate salts are the most common mineral containing phosphorus and are less available, although they can also dissolve under certain pH conditions^4,33,57^. Liu et al.^58^ observed the highest phosphate removal efficiency from solutions using calcium carbonate already in the pH range of 6–7. When using aluminium sulphate, buffering with sodium aluminate is often used to eliminate large pH changes ^7^. Łopata et al.^59^ showed that the addition of calcium carbonate effectively buffered the decrease in water pH caused by the addition of polyaluminium chloride and reduced the amount of dissolved Al in the water, particularly when high doses of coagulant were used. Treatment of water with calcium does not cause the breakdown of phytoplankton cells, so it may be an effective and safe method to control cyanobacterial blooms^60^. The biotoxicity of iron and calcium is much lower compared to other metallic elements such as aluminium, manganese, and rare earth elements^9,17,46^. Despite the very low content of P_tot._ and OM, the sandy sediments of the Brzóza Królewska reservoir in the dam area (BK2) required relatively high doses of the mixture. This may indicate the formation of humic-Fe/Ca-phosphorus complex connections as one of the mechanisms of phosphorus binding by the substances in the mixture in the case of the other sediments. The presence of such connections in the sediments of the studied reservoirs was confirmed by the appearance of statistically significant correlations between OP and humic fractions^51^. According to Lürling et al.^61^, humic substances can play an important role in reducing the effectiveness of metal-based phosphorus sorbents. They observed a strong increase in the dissolved lanthanum in water in the presence of humic substances, implying a limitation in the use of La-modified bentonite in inland waters rich in dissolved organic carbon. Huang et al.^15^ also observed that the presence of humic acids (HA) in solution reduced the phosphate adsorption capacity of the La-modified material by approximately 30%, mainly because a complex compound of La-HA was formed, which hindered the binding of phosphate and La. There was also information that humic acids competed with phosphates for sorption sites on aluminium hydroxide flocs ^23^. However, phosphate anions and humic substances can form stable and poorly water-soluble complexes by bridging with a metal cation. In particular, trivalent metals such as Fe(III) and Al(III) tend to form strong covalent bonds with HS. Divalent metals (e.g. Ca) can also bind humic substances to phosphates ^3,5^. Through the formation of stable connections of the humic-Fe(III)-phosphate complex, the reducible Fe was partially stabilised in the humic-rich bottom sediments ^3^. According to Junakova et al. ^16^, the size of the sediment particles, their specific surface, the content and nature of the organic matter, and the content of aluminium and iron in the sediment were the main factors affecting the retention of phosphorus in the sediment. A slightly weaker efficiency of the tested mixture was also observed for the more hydrated sediments of the Nowa Wieś reservoir in the dam area (NW2) after a 10-week exposure. Significant sediment hydration can limit the inactivating effect of the mixture by dropping its components into deeper sediment layers below the zone actively involved in the exchange process.

Significant phosphorus loads were still released as a result of repeated resuspension. For ecological safety reasons, the concentration of total phosphorus in the water should be controlled to be within the range that does not cause phytoplankton blooms. To maintain control over trophic degradation, the concentration of total phosphorus in reservoir waters should occur in the range of 0.005 to 0.1 mgP∙dm^-3^ ^62^. P concentrations already > 0.02 mgP∙dm^-3^ usually accelerate lake eutrophication ^17^. Vuorio et al. ^63^ based on studies of 2,000 Finnish lakes (considering all types of cyanobacteria and lake types), showed that the TP concentration of 0.03 mgP∙dm^-3^ can be considered a critical value for inducing cyanobacterial blooms. Thus, in order to achieve more stable reclamation effects, the sediments will require the application of the salt mixture in the subsequent years. This activity is practised for other substances and reactive materials used in the chemical reclamation of lakes ^20^. The potential for internal P loading is likely to increase in the future, due to climate change in temperate regions^1,13,47,64^, as shallow water reservoirs in particular are warming.

## Conclusions

The main determinant of the advanced degradation of water reservoirs is the presence of intensive internal supply. Excess phosphorus in the water must be eliminated to control the progress of trophic degradation and maintain a sustainable environment for future generations. A key factor in small retention reservoirs that determines the occurrence of internal phosphate supply is the significant enrichment of bottom sediments in organic matter and mobile forms of phosphorus. The synergy of the CaSO_4_∙2H_2_O, FeCl_3_ and CaCO_3_ salts was ideal to inactivate phosphate phosphorus in the bottom sediments of the reservoirs studied. Although the use of Fe-containing materials does not cause ecotoxicological effects, water acidification can be a problem. FeCl_3_ and CaCO_3_ act as pH buffering agents for each other and their application in certain proportions does not lead to a significant increase or decrease in water pH. The mixture of calcium and iron salts showed good effectiveness (approximately 100%) under anoxic conditions at maximum doses in the range 0.23–0.41 g. Increasing the exposure time to 24 h without resuspension of the sediment had no significant effect on the amount of phosphorus released. The decisive influence on the total internal phosphorus load is the release under dynamic conditions, that is, during simulated sediment resuspension and suspension resedimentation. Due to the weaker effectiveness of the action in long-term studies, it will be advisable to repeat the application of these substances in subsequent years or several times a year starting in early spring during the reclamation of water reservoirs. The applied salt mixture showed weaker effectiveness in sandy sediments (78–93% sand), poor in organic matter (1–6.5% OM), especially humic substances (2.8–29 mgC∙g^-1^ dw), and significantly hydrated sediments (71% water). Despite these limitations, the tested salt mixture can be successfully used in the "flock and lock" technique as a material that effectively inactivates phosphorus.

## Data Availability

The authors declare that the data supporting the findings of this study are available within the paper. Should any raw data files be needed in another format they are available from the corresponding author upon reasonable request. Source data are provided with this paper. Example from https://doi.org/10.1007/s00027-023-01003-4
